# Epidemiologic characteristics of scrub typhus on Jeju Island

**DOI:** 10.4178/epih.e2017039

**Published:** 2017-08-18

**Authors:** Sung Uk Lee

**Affiliations:** Jeju Special Self-Governing Provincial Office, Jeju, Korea

**Keywords:** Scrub typhus, Rickettsia, Incidence, Republic of Korea

## Abstract

**OBJECTIVES:**

Scrub typhus is the most common febrile disease in Korea during the autumn. Jeju Island is the largest island in South Korea and has a distinctive oceanic climate. This study aimed to identify epidemiologic characteristics of scrub typhus on Jeju Island.

**METHODS:**

From January 2011 to December 2016, 446 patients were diagnosed with scrub typhus on Jeju Island. The patients’ personal data and the environmental factors that might be related to scrub typhus were investigated and retrospectively analyzed.

**RESULTS:**

The median age of the patients was 58-years-old (range, 8 to 91) and 43% of them worked in the agricultural, forestry or livestock industry. Regardless of their job, 87% of the patients had a history of either working outdoors or of other activities before developing scrub typhus. The south and southeast regions of Jeju Island, especially Namwon-eup, showed the highest incidence of scrub typhus. Workers in mandarin orange orchards seemed to be the highest risk group for scrub typhus infection.

**CONCLUSIONS:**

Scrub typhus on Jeju Island showed unique characteristics. To efficiently prevent scrub typhus, each year individual regional approaches should be developed based on the epidemiologic characteristics of the disease.

## INTRODUCTION

Scrub typhus is a mite-borne infection that belongs to the Group III notifiable infectious diseases; it is caused by the bacterium *Orientia tsutsugamushi* [1]. The vector of this bacterium is larval trombiculid mites or chigger mites of the genus *Leptotrombidium* [2]. Chigger mites must feed on the tissue fluid of a host animal during their transformation from larvae to adults. At this time, when a larva is accidently transferred to a person and feeds on their tissue fluid, the causative agent is transmitted through the host’s skin, infecting the individual [3]. Scrub typhus is a typical febrile disease during the autumn. After an average incubation period of 10- 12 days, the onset of scrub typhus is characterized by symptoms such as high fever, myalgia and skin rash. An eschar that is formed in the bite sites of the chigger mites is a well-known clinical feature of patients with scrub typhus. Scrub typhus is endemic to Asia-Pacific tropical and rural areas including South Korea (hereafter Korea), Japan, and India, which are called ‘tsutsugamushi triangle’, and South-East Asia [4]. In Korea, an increasing trend in the incidence of scrub typhus has been evident every year since 2009. In 2016, 11,105 patients with scrub typhus were reported with an increase of 16.7% over the previous year [5].

Located on the southernmost tip of Korea, Jeju Island is the largest island in Korea, occupying 1.83% of the total area of Korea, and its population was about 620,000 as of 2015 (Table 1) [6]. In terms of general climate classification, Jeju Island has a latitude range of about 33-34 degrees, and is located in a transition zone ranging from a subtropical climate to temperate climate. Because Jeju Island is surrounded by the sea, it has a warm and wet oceanic climate throughout the year with higher temperature and precipitation compared to the inland regions of the Korean peninsula [7]. The incidence of scrub typhus tends to be higher in the southern region than that in the northern region in the inland regions of the Korean peninsula; however, it tends to decrease again on Jeju Island [5]. Studies on scrub typhus conducted in Korea and foreign countries have been mainly focused on case reports, clinical findings, diagnoses or serological studies and treatments. However, epidemiological studies investigating systemic occurrence patterns and risk factors of scrub typhus are scarce. In particular, little is known about the epidemiologic characteristics of scrub typhus occurring on Jeju Island. Therefore, the present study aimed to identify the unique characteristics of scrub typhus on Jeju Island.

## MATERIALS AND METHODS

### Subjects

We analyzed the cases of scrub typhus investigated by the Department of Health and Sanitation, Jeju Special Self-Governing Province from 2011 to 2016. During this time the number of patients with scrub typhus in the Jeju Special Self-Governing Province was 446 with 57 in 2011, 72 in 2012, 52 in 2013, 55 in 2014, 61 in 2015, and 149 in 2016. They were all serologically or clinically diagnosed with scrub typhus, and included in the infectious disease surveillance web statistics of the Korea Centers for Disease Control and Prevention (KCDC). Each case report of scrub typhus was prepared by public health officers in charge of infectious diseases in 6 regional health centers (Jeju Health Center, Jeju East Health Center, Jeju West Health Center, Seogwipo Health Center, Seogwipo East Health Center, Seogwipo West Health Center). Jeju Special Self-Governing Province consists of administrative districts including 2 cities, 7 ‘eups’ and 4 ‘myeons’. The 6 regional health centers are responsible for the following areas: the Jeju Health Center for Jeju-si East; the Juju East Health Center for Jocheon-eup, Gujwa-eup, and Udo-myeon; the Juju West Health Center for Aewoleup, Hallim-eup, and Hangyeong-myeon; the Seogwipo Health Center for Seogwipo-si East; the Seogwipo East Health Center for Seongsan-eup, Pyoseon-myeon, and Namwon-eup; and the Seogwipo West Health Center for Daejeong-eup and Andeok-myeon (Table 1).

### Methods

Data on the occurrence, year, and location of the 446 patients with scrub typhus on Jeju Island were obtained from the infectious disease surveillance web statistics of the KCDC, and the incidence of scrub typhus by year and region was investigated. The total population of Jeju Island and the population by region were based on the average population from 2011 to 2015 from the National Statistics Office data in Korea. In order to divide the administrative districts into urbanized areas and rural areas, they were divided into ‘dong’ areas and ‘eup’-‘myeon’ areas, respectively. The case reports contained basic personal information such as sex, age, address, occupation, and disease-related factors (date of onset, type of work, experience of recent outdoor activities, and clinical symptoms). In order to investigate the relationship between scrub typhus and occupation, the occupation-related exposures of the subjects were divided into four categories: 1) those working in the agriculture, forestry or livestock industry; 2) other outdoor workers; 3) those engaged in occupation-unrelated one-time or hobby agriculture, forestry or livestock work (including vegetable gardening, weekend gardening); and 4) those engaged in simple outdoor activities (including hiking, picnic). In order to analyze the risk factors for scrub typhus infection in the case reports, the types of outdoor work and activities performed by subjects were analyzed; these activities were also analyzed to determine whether the 6 regional public health centers demonstrated different characteristics.

### Data analysis

All data were electronically recorded. Statistical testing was performed using SPSS version 23 (IBM SPSS Inc., Chicago, IL, USA). The Mann Whitney U-test was used as a non-parametric measure for comparing 2 independent groups. For the Pearson’s chi-square test p< 0.05 was considered statistically significant to determine if there was a difference between discontinuous variables consisting of an r× c table.

## RESULTS

### Demographic characteristics

This epidemiologic investigation consisted of 446 subjects who were followed for 6 years; there were 201 males (45.0%) and 245 females (54.9%). The median age was 58 years (range, 8 to 91 years). By age group, the number of the subjects aged younger than 20, 20-39, 40-59, 60-79, and 80 years or older was 5 (1%), 48 (11%), 187 (42%), 176 (39%), and 30 (7%), respectively. There were 318 subjects (71%) who were diagnosed with scrub typhus confirmed by laboratory tests and 128 subjects (29%) who were clinically diagnosed. The distribution of occupation-related exposure history showed 193 subjects (43%) working in the agriculture, forestry or livestock industry; 45 (10%) outdoor workers other than the agriculture, forestry or livestock industry; 79 subjects (18%) engaged in occupation-unrelated one-time or hobby agriculture, forestry or livestock work (vegetable gardening, weekend gardening); 71 subjects (16%) engaged in simple outdoor activities; and 58 subjects (13%) without a history of specific outdoor activity.

### Regional incidence of scrub typhus in Jeju Special Self-Governing Province (2011-2016)

**Figure 1.**
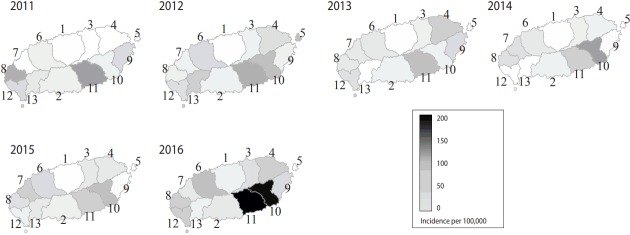
Regional incidence of scrub typhus by year (2011-2016). 1, Jeju-si; 2, Seogwipo-si; 3, Jocheon- eup; 4, Gujwa-eup; 5, Udo-myeon; 6, Aewol-eup; 7, Hallim-eup; 8, Hangyeong-myeon; 9, Seongsan-eup; 10, Pyoseon-myeon; 11, Namwon-eup; 12, Daejeong-eup; 13, Andeokmyeon.

Among the total population in Jeju from 2011 to 2016, the 446 patients with scrub typhus represented an incidence of 9.5 per 100,000 in 2011, 12.1 per 100,000 in 2012, 8.7 per 100,000 in 2013, 9.2 per 100,000 in 2014, 10.2 per 100,000 in 2015, and 25.0 per 100,000 persons in 2016. The number of patients with scrub typhus and the incidence of scrub typhus categorized by each of the 6 regional health centers are presented in Table 2. There was a statistically significant difference in the incidence of scrub typhus per 100,000 persons between Jeju-si and Seogwipo-si among the urbanized ‘dong’ area regions (p= 0.006). When comparing the incidence of scrub typhus per 100,000 persons in 2011-2016 between the rural ‘eup’-‘myeon’ areas, there was no statistically significant difference in the incidence of scrub typhus per 100,000 persons between Jeju East and Jeju West regions (p= 0.26), but there was a significant difference between Seogwipo East and Seogwipo West regions (p= 0.004). Looking at the annual incidence of scrub typhus per 100,000 persons in 2 cities, 7 ‘eup’ and 4 ‘myeon’ areas in Jeju Special Self-Governing Province, the annual incidence of scrub typhus from 2011 to 2016 was high in Namwoneup and Pyoseon-myeon as the south and southeast regions of Jeju Island or Seogwipo East region, and the annual incidence of scrub typhus was less than 10 per 100,000 persons in the urban areas of Jeju Island (Figure 1). Namwon-eup in Seogwipo showed the highest incidence of scrub typhus with 197.8 per 100,000 persons in 2016 (Table 2).

### Temporal characteristics

**Figure 2.**
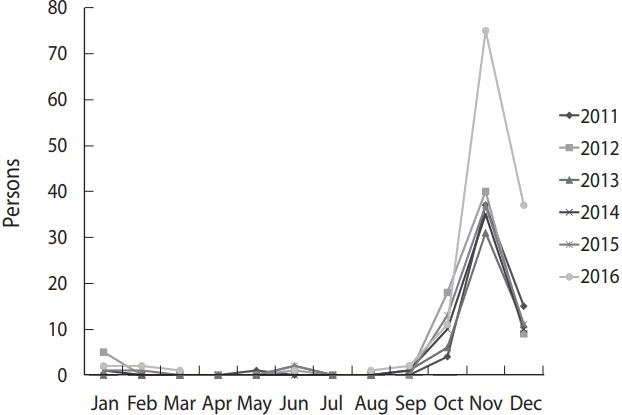
Monthly trend of scrub typhus. The incidence peaked in November.

The monthly distribution of the incidence of scrub typhus from 2011 to 2016 showed that less than 2 cases of scrub typhus occurred from February to September, and then the number of cases greatly increased from October every year with a similar curve distribution throughout the overall period (Figure 2). Of the total number of the subjects with scrub typhus from 2011 to 2016 (n= 446), 62 (14%) were reported in October, 255 (57%) in November, 93 (21%) in December, and 9 (2%) in January.

### Risk factor analysis

Of the 446 subjects with scrub typhus from 2011 to 2016, 388 subjects (87%) who were reported to have had outdoor activities that could have exposed them to the mites during the incubation period were classified for their detailed activities (Table 3). Of the outdoor activities, there were 155 subjects (35%) who were engaged in fruit farming or had a history of one time working in an orchard, 103 subjects (23%) who worked professionally or one time in dry field farming, 68 subjects (15%) who had a history of simple outdoor activities such as hiking and walking, 24 subjects (5%) who had a history of working outdoors mowing or pulling weeds or other like activities, 16 subjects (4%) who had a history of contact with livestock such as cattle and horses, 6 subjects (1%) who had a history of wild fern and herb gathering, 6 subjects (1%) who were engaged in forestry, and 3 subjects (1%) who worked on a golf course. The types of crops were identified in 109 of the 155 subjects who worked in fruit farming; the crop work consisted of 99 subjects (91%) in mandarin orange orchard farming, 4 subjects (4%) in persimmon farming, and 3 subjects (3%) in kiwi farming.

In each of the 6 regional health centers on Jeju Island the distribution of the subjects with possible exposure to mites was summarized (Table 3). The subjects who had a history of exposure to the top 3 outdoor activities (working in orchards, dry field farming, and simple outdoor activities) with a high proportion of detailed activities were classified by each of the 6 regional health centers. As a result, there was a significant difference in the distribution of outdoor exposures between public health centers (p< 0.001). The most frequent outdoor exposure was simple outdoor activities in Jeju East region, working in orchards in Seogwipo-si ‘dong’ area and Seogwipo East region, and dry field farming in Jeju East and Jeju West regions and Seogwipo West region.

## DISCUSSION

The present study aimed to analyze 446 patients with scrub typhus that occurred on Jeju Island 2011 to 2016, to understand the regional, temporal and demographic characteristics of the affected subjects, and to identify risk factors for scrub typhus. According to the known characteristics of affected patients, scrub typhus was found to occur more frequently in females. In age distribution, adults aged 50 or older comprised the vast majority of cases, and the scrub typhus predominantly occurred in individuals who worked in agriculture, helped with the harvest, and those who hiked or gathered fruits and seeds such as acorns [8]. The sex ratio of cases in Jeju Island was 45% males and 55% females with a higher incidence in females; however, the incidence gap between the two sexes was narrower than the 35% in males and 65% in females previously reported by an inland epidemiologic study [2]. The finding that scrub typhus occurred more frequently in those aged 50-60 engaged in agriculture in October and November, or during the autumn crop harvest season, was similar to the results of the inland epidemiologic study. However, the unique point was that the infection rate was the highest while working in orchards, and the main related work was mandarin orange farming. This is different from that in inland regions where dry field work is the main route of infection [9]. The results of the present study showed that orchard farming, which is representative of mandarin oranges on Jeju Island, was a main risk factor for scrub typhus in Jeju Island unlike inland regions. The harvest time of mandarin oranges, from October through December, is consistent with the epidemic of scrub typhus. Further studies regarding the life cycle of chigger mites are needed to determine what kinds of behaviors may increase the risk of scrub typhus infection during mandarin orchard work.

When detailed activities related to exposure to mites were separated by regional public health center, the results indicated that the distribution of exposure to mites differed according to each region. Fruit farming was the main putative infection route in Seogwipo-si ‘dong’ area and Seogwipo East region, whereas dry field farming or simple outdoor activities were the main putative infection routes in the other regions. This is related to the fact that the cultivation status of crops in Jeju Island varies regionally according to soil and climate characteristics. Seogwipo-si ‘dong’ area and Namwon-eup and Pyoseon-myeon in Seogwipo East region, as the south and southeast areas of Jeju Island, are the chief producing areas for mandarin oranges, and are dominated by fruit farming. The rest of the region is dominated by dry field farming with its main crops being root vegetables, western vegetables, or grains; Jeju East region is an urbanized area occupied with tertiary industries such as service industries rather than primary industries [10]. Based on this, it is necessary to develop customized plans to prevent scrub typhus specific to the main infection route in each regional health center. Meanwhile, it can be assumed that the reason for the higher incidence of scrub typhus in Seogwipo-si ‘dong’ area and Seogwipo East region might be related to fruit farming. Looking at regional Agricultural Cooperative mandarin orange shipments on Jeju Island in 2015, 78.8% of Agricultural Cooperative mandarin shipment on Jeju Island were reported to have been shipped from Seogwipo-si ‘dong’ area and Namwon-eup in Seogwipo East region [11]. In support of this, an in-depth epidemiological study report in 2006 published by the KCDC demonstrated that agricultural fruit farming significantly increased the risk of developing scrub typhus compared to the other kinds of agriculture [2].

In particular, of the analyzed period, 2016 was the year with a high incidence of scrub typhus. The incidence of scrub typhus increased from the preceding year in most regions (excluding Hallim-eup, Andeok-myeon) in Jeju Special Self-Governing Province, and the incidence rates of scrub typhus in Namwon-eup and Pyoseon-myeon soared to 197.8 and 181.8 per 100,000 persons, respectively. In 2016, the average incidence of scrub typhus nationwide was reported to be 21.5 per 100,000 persons, and most frequently occurred in three areas nationwide, Jeonnam with 92.5 per 100,000 persons, Gyeongnam with 69.4, and Jeonbuk with 55.1 [5]. First, in 2016, the number of reported cases of Group III notifiable infectious diseases including scrub typhus increased by 39% as a result of enhanced awareness of notifiable infectious disease reporting and the reorganization of the national quarantine system due to the Middle East Respiratory Syndrome incident in the preceding year [5]. In addition, the KCDC estimates that the average temperature in August, which is the spawning season of chigger mites in Korea, is a factor affecting the density of chigger mites [9]. This is because in summer (especially, August) when temperature is high and the weather is relatively dry, grassy vegetation becomes lush and the growth and breeding of chigger mites as the scrub typhus vector is also active. In August of 2016 on Jeju Island, the average temperature substantially rose to 28.1°C due to the influence of the thermal high pressure developed in the North Pacific and the Chinese continent that was recorded as the 7th highest average temperature since 1961 [12]. This is thought to have contributed to an increase in the incidence of scrub typhus.

In order to achieve substantial results with limited resources, infection prevention management projects should be developed based on the epidemiological characteristics of the related infectious diseases; selective, focused strategies suited to the regional characteristics of the related infectious disease are also needed. The results of the present study found that mandarin orange orchard farming was a major risk factor for scrub typhus. The exposure to risk factors was found to be different according to each region on Jeju Island, and thus it is necessary to establish customized preventive measures specific to Jeju Island.

## Figures and Tables

**Table 1.** Brief regional demographics of Jeju Island

	Regions	Area	Total area (km^2^)	2011-2015 average population (10^3^, %)	Population density (person/km^2^)	Main industry^1^
City	Jeju	Jeju-si	264	343 (58)	1,301	Tertiary
	Seogwipo	Seogwipo-si	254	87 (15)	343	Mix (primary +tertiary)
Countryside	Jeju East	Gujwa-eup	185	15 (2)	79	Primary
		Jocheon-eup	151	21 (4)	136	
		Udo-myeon	6	2 (<1)	333	
	Jeju West	Aewol-eup	202	30 (5)	147	Primary
		Hallim-eup	91	20 (3)	215	
		Hangyeong-myeon	79	8 (1)	104	
	Seogwipo East	Seongsan-eup	108	14 (2)	130	Primary
		Pyoseon-myeon	135	11 (2)	81	
		Namwon-eup	189	18 (3)	96	
	Seogwipo West	Daejeong-eup	79	17 (3)	218	Primary
		Andeok-myeon	106	10 (2)	94	

1 Primary industry includes farming, livestock and fishing; Tertiary industry includes retailing, tourism and administrative service.

**Table 2.** Regional incidence^1^ of scrub typhus in Jeju Island

	Regions	2011		2012		2013		2014		2015		2016		p-value^2^
		Case (n)	Incidence	Case (n)	Incidence	Case (n)	Incidence	Case (n)	Incidence	Case (n)	Incidence	Case (n)	Incidence	
City	Jeju-si	9	2.6	16	4.7	13	3.8	14	4.1	6	1.7	34	9.9	0.006
	Seogwipo-si	12	13.8	10	11.5	8	9.2	9	10.3	13	14.9	16	18.4	
Countryside	Jeju East	1	2.7	7	18.8	8	21.5	4	10.8	7	18.8	10	26.9	0.26
	Jeju West	10	17.4	13	22.6	8	13.9	11	19.2	18	31.4	21	36.6	
	Seogwipo East	18	41.7	18	41.7	13	30.1	16	37.0	13	30.1	60	138.9	0.004
	Seogwipo West	7	25.7	8	29.4	2	7.4	1	3.7	4	14.7	8	29.4	

1 Per 100,000 persons.

2 Calculated by Mann Whitney U-test for the incidence.

**Table 3.** Exposure that may cause scrub typhus by region

	Regions	Total (n)	Exposure				p-value^2^
			Orchard	Dry field	Hiking	Others^1^	
City	Jeju	76	15 (20)	18 (24)	26 (34)^3^	17 (22)	<0.001
	Seogwipo	57	34 (60)^3^	8 (14)	10 (18)	5 (9)	
Countryside	Jeju East	34	7 (21)	13 (38)^3^	8 (24)	6 (18)	
	Jeju West	67	14 (21)	28 (42)^3^	13 (19)	12 (18)	
	Seogwipo East	117	76 (65)^3^	20 (17)	9 (8)	12 (10)	
	Seogwipo West	30	9 (30)	16 (53)^3^	2 (7)	3 (10)	

Values are presented as number (%).

1 Others include livestock, forestry, mowing and herb gathering.

2 Calculated by Pearson chi-square test

3 The most common exposure in each region.
