# Supplementary material for: Epidemiologic characteristics of scrub typhus on Jeju Island
Source: Epidemiol Health. 2017 Aug 18;39:e2017039. doi: 10.4178/epih.e2017039 (PMC5675983; doi:10.4178/epih.e2017039)
Supplement: Supplementary file 1 [file epih-39-e2017039-supplementary.pdf]

Volume: 39, Article ID: e2017039
https://doi.org/10.4178/epih.e2017039

제주도 쯔쯔가무시증의 역학적 특성

Epidemiologic characteristics of scrub typhus in Jeju Island

이성욱[1]

[1]제주특별자치도청 보건위생과

Sung Uk Lee[1]

[1]Division of Health and Sanitation, Jeju Special Self-Governing Provincial Office

Corresponding author: Sung Uk Lee, Epidemiologic intelligence service officer

Address: 6, Munyeon-ro, Jeju-si, Jeju special self-governing province, Republic of Korea, 63122

E-mail : sulee86@korea.kr

Volume: 39, Article ID: e2017039
https://doi.org/10.4178/epih.e2017039

Abstract

OBJECTIVES

Scrub typhus is the most common febrile disease in Korea during the autumn. Jeju Island is the largest island in South Korea and has a distinctive oceanic climate. This study aimed to identify epidemiologic characteristics of scrub typhus on Jeju Island.

METHODS

From January 2011 to December 2016, 446 patients were diagnosed with scrub typhus on Jeju Island. The patients' personal data and the environmental factors that might be related to scrub typhus were investigated and retrospectively analyzed.

RESULTS

The median age of the patients was 58-years-old (range, 8 to 91) and 43% of them worked in the agricultural, forestry or livestock industry. Regardless of their job, 87% of the patients had a history of either working outdoors or of other activities before developing scrub typhus. The south and southeast regions of Jeju Island, especially Namwon-eup, showed the highest incidence of scrub typhus. Workers in mandarin orange orchards seemed to be the highest risk group for scrub typhus infection.

CONCLUSIONS

Scrub typhus on Jeju Island showed unique characteristics. To efficiently prevent scrub typhus, each year individual regional approaches should be developed based on the epidemiologic characteristics of the disease.

KEY WORDS: Scrub typhus, Rickettsia, Incidence, Republic of Korea

Volume: 39, Article ID: e2017039
https://doi.org/10.4178/epih.e2017039

INTRODUCTION

쯔쯔가무시증은 제3군 법정 전염병에 속하는 진드기 매개질환으로 *Orientia tsutsugamushi* 에 의하여 발생하는 질환이다 [1]. 이 균을 매개하는 것은 털진드기과 진드기 유충인 chigger mite 중 *Leptotrombidium* 속 털진드기이다 [2]. 털진드기가 유충에서 성충으로 탈바꿈할 때, 반드시 동물의 조직액을 섭취하여야 하는데 이 시기에 우연히 유충이 사람에게 옮겨져 조직액을 섭취할 때 병원체가 피부를 통해 침입함으로써 감염되게 된다 [3]. 쯔쯔가무시증은 대표적인 가을철 발열성 질환으로 평균 10-12일의 잠복기가 지난 이후에 고열, 근육통, 피부발진 등의 증상 보이며, 털진드기 유충이 물린 자리에 형성되는 가피는 쯔쯔가무시증 환자의 잘 알려진 임상적 특징이다. '쯔쯔가무시 삼각형 (tsutsugamushi triangle)' 지역이라 불리는 한국, 일본, 인도와 동남아를 포함하는 아시아 태평양의 열대 및 농촌 지역에서 많이 발생하고 있는데 [4]., 최근 국내에서는 2009년 이후로 매년 증가 추세이며 2016년도에는 전년대비 16.7% 증가한 11,105명의 환자가 보고되었다[5].

대한민국 최남단에 위치하고 있는 제주도는 대한민국에서 가장 큰 섬으로 대한민국 면적의 1.83%에 해당하며, 인구는 2015년 기준으로 62만여명이다 (Table 1)[6].

제주도는 위도 범위가 약 33-34도로 일반적인 기후 분류상 아열대기후대에서 온대기후대로의 전이지대에 위치하고 있으며, 사면이 바다이므로 연중 온난 습윤한 해양성이 강하여 한반도 내륙지방의 값과 비교하면 기온이 높고 강수량이 많다 [7]. 한반도 내륙지역에서는 남부지역으로 갈수록 쯔쯔가무시증 발생률이 북부지역보다 높아지는 경향이 있지만 제주 지역에서는 다시 낮아 지는 양상을 보인다 [5]. 국내외에서 이루어진 쯔쯔가무시증에 대한 연구는 주로 환자사례보고를 비롯한 임상적 소견, 진단 혹은 혈청학적 연구, 치료법에 관한 내용이 주를 이루고, 체계적인 발생 양상이나 수준, 발병 위험요인을 밝히고자 하는 역학 연구는 거의 이루어진 바 없다. 특히 제주 지역에서 발생하는 쯔쯔가무시증의 역학적 특징에 대해서 알려진 바가 적어, 본 연구를 통해 제주 지역만의 특수성이 있는지 살펴보고자 한다.

METHODS AND MATERIALS

1. 연구대상

2011년부터 2016년까지 제주특별자치도 보건위생과에서 조사된 쯔쯔가무시증 사례들을 연구 대상으로 하여 분석하였다. 제주특별자치도에서 2011년부터 2016년까지 발생한 쯔쯔가무시증 환자는 2011년에 57명, 2012년에 72명, 2013년에 52명,

2014년에 55명, 2015년에 61명, 2016년에 149명으로 총 446명 이었다. 이들은 모두 혈청학적 또는 임상적으로 진단되었고, 질병관리본부의 감염병 웹통계에 포함된 환자들이다. 쯔쯔가무시증 개별 사례조사서는 제주특별자치도 내 6개 지역 보건소 (제주 보건소, 제주 동부보건소, 제주 서부보건소, 서귀포 보건소, 서귀포 동부보건소, 서귀포 서부 보건소) 의 감염병 담당자에 의해 작성되었다. 제주특별자치도는 2개 시, 7개 읍, 3개 면의 행정구역으로 이루어져 있고, 6개 지역 보건소가 담당하는 지역은 제주시보건소는 제주시 동지역을, 제주동부보건소는 조천읍, 구좌읍, 우도면을, 제주서부보건소는 애월읍, 한림읍, 한경면을, 서귀포시보건소는 서귀포시 동지역을, 서귀포동부보건소는 성산읍, 표선면, 남원읍을, 서귀포서부보건소는 대정읍과 안덕면으로 나누어 담당하고 있다 (Table 1).

2. 연구방법

질병관리본부의 감염병 웹통계에서 제주특별자치도에서 발생한 쯔쯔가무시증 환자 446명의 발생연도 및 발생지역에 관한 자료를 확보하여 연도별, 지역별 발생 현황을 파악하였다. 제주도 전체 인구 및 지역별 인구는 통계청 자료 중 2011년부터 2015년 까지의 평균 인구를 적용하였다. 행정구역 중 도시화된 지역과 시골

Volume: 39, Article ID: e2017039
https://doi.org/10.4178/epih.e2017039

지역을 나누기 위해 동지역과 읍면지역으로 구분하였다. 사례조사서에는 성별, 나이, 주소, 직업 등의 기본 인적 사항, 발병 일자, 작업 종류, 최근 야외 활동 경험 유무 등의 질병 발생 관련 요인과 임상 증상 관련 정보 등이 포함되어 있었다. 쯔쯔가무시증과 직업과의 연관성을 보기 위해, 대상자의 직업 관련 노출력을 농림축산업 종사자, 농림축산업에 외 야외 작업 종사자, 직업과 관련 없는 일회성 혹은 취미성 농림축산업 (텃밭, 주말농장 포함)과 단순 야외 활동 (등산, 소풍 포함)의 4개의 카테고리로 나누었다. 사례조사서 내용 중 쯔쯔가무시증 감염의 위험 요인 분석을 위해 대상자 별 야외 작업 및 활동의 종류에 대하여 분석하고 6개 지역 보건소 별로 다른 특성이 있는지 분석하였다.

3. 자료 분석

모든 자료는 전산 입력 하였고, 통계검정은 SPSS statistic version 23 (SPSS, Chicago, IL, USA)을 이용하였다. 독립된 2개군의 비교를 위한 비모수적 방법으로 Mann Whitney U-test를 이용하였고, r x c table로 구성된 비연속 변수간의 유의한 차이가 있는지 분석하기 위해 Pearson’s chi-square test 를 이용하여 $p<0.05$를 유의한 것으로 분석하였다.

Volume: 39, Article ID: e2017039
https://doi.org/10.4178/epih.e2017039

RESULTS

1. 인적 특성

6년간 역학조사가 된 대상자 446명의 성별 분포는 남자 201명 (45.0%), 여자 245명 (54.9%) 이었다. 연령 중앙값은 58세(8-91세) 이었고 연령별 분포는 20세 미만이 5명(1%), 20-39세가 48명(11%), 40-59세가 187명(42%), 60-79세가 176명(39%), 80세 이상이 30명(7%) 이었다. 실험실적 검사법으로 확진 받은 대상자는 318명(71%), 임상적 진단 받은 대상자는 128명 (29%) 이었다. 대상자 직업 관련 노출력의 분포는 농림축산업 종사자 193명 (43%), 농립축산업 외 야외 작업 종사자 45명 (10%), 직업과 관련 없는 일회성 혹은 취미성 농림축산업 (텃밭, 주말농장 포함)이 79명 (18%), 단순 야외 활동 71명 (16%) 외 특별한 야외 노출력 없음 58명 (13%) 으로 조사 되었다.

2. 제주특별자치도 내 지역별 발생 현황 및 발생률 (2011-2016)

제주특별자치도 내 2011년부터 2016년까지 발생한 446명의 제주도 전체 인구에 대한 발생률은 인구 10만명당 2011년에 9.5명, 2012년에 12.1명, 2013년에 8.7명, 2014년에 9.2명, 2015년에 10.2명, 2016년에 25.0명 이었고 6개 지역 보건소별 환자

발생수 및 발생률은 Table 2에 기술하였다. 도시화된 동지역 중에서 제주시와 서귀포시 지역의 2011-2016년 10만명당 발생률은 통계적으로 유의한 차이를 보였다 (p=0.006). 시골지역인 읍면지역간의 2011-2016년 10만명당 발생률을 비교해 보았을 때, 제주 동부 지역과 서부 지역은 통계적으로 유의한 차이를 보이지 않았으나 (p=0.261), 서귀포 동부 지역과 서부 지역의 발생률은 유의한 차이를 보였다 (p=0.004). 제주특별자치도의 2개 시, 7개 읍, 3개 면의 행정구역 연도별 10만명당 쯔쯔가무시증 발생률을 살펴보면 제주도의 남•남동권이자 서귀포 동부권인 남원읍과 표선면 지역에서 2011년부터 2016년 까지 매년 높은 발생률을 보였고, 제주 지역의 도심지역에서는 10만명당 발생률이 매년 10명 미만으로 관찰되었다 (Figure 1). 서귀포의 남원 지역에서는 2016년 10만 명당 197.8명의 가장 높은 발생률을 보였다 (Table 2).

3. 시간적 특성

2011년부터 2016년까지 전체 발생월별 분포는 2월부터 9월까지는 매년 2건 이하로 발생하다 10월부터 크게 상승하는 양상으로 전체적으로 유사한 곡선 분포를 보였다 (Figure 2). 2011년부터 2016년까지 전체 발생 대상자수 (n=446) 중 10월 발

Volume: 39, Article ID: e2017039
https://doi.org/10.4178/epih.e2017039

생 대상자수는 62명 (14%), 11월은 255명 (57%), 12월은 발생 대상자수 93명 (21%), 1월은 9명 (2%) 보고 되었다.

4. 위험요인 분석

2011년부터 2016년 까지 쯔쯔가무시증 대상자 총 446 명 중 잠복기 내 진드기에 노출될 수 있는 야외 활동이 있었던 것으로 조사된 388명 (87%) 의 상세 활동 종류에 대해 분류 하였다 (Table 3). 야외 활동 중 과수업 종사자 혹은 일회성 과수원 작업력이 있는 자 155명 (전체 대상자 중 35%), 직업적 혹은 일회성 논•밭농사 작업력이 있는 자 103명 (23%), 등산•산책 등의 단순 야외 활동력이 있는 자 68명 (15%), 풀베기•풀뽑기 등의 수풀 관련 작업력이 있는 자 24명 (5%), 소•말 등의 가축 접촉력 16명 (4%), 야산 고사리•약초 채취 6명 (1%), 임업 종사자 6명 (1%), 골프장 종사자 3명 (1%) 순으로 조사되었다. 과수업 종사자 155명 중 109명에서 작물 종류가 조사되었는데 감귤 99명 (91%), 감 4명 (4%), 키위 3명 (3%) 순이었다.

제주특별자치도 6개 지역 보건소 별 대상자의 진드기 노출력 분포를 정리하였다 (Table 3). 조사된 상세 활동 중 높은 비율을 보인 3대 야외 노출력 (과수원 작업,

Volume: 39, Article ID: e2017039
https://doi.org/10.4178/epih.e2017039

논•밭 작업, 단순 야외 활동)이 있는 대상자를 6개 지역 보건소 별로 분류해 보았을 때, 보건소 별로 통계적으로 유의하게 다른 분포를 보였다 (p=0.000). 가장 높은 빈도의 야외 노출력이 제주 동지역은 단순 야외활동, 서귀포 동지역과 서귀포 동부 지역은 과수원 작업, 제주 동서부 지역, 서귀포 서부 지역은 논•밭 작업인 것으로 차이를 보였다.

DISCUSSION

본 연구에서는 2011년부터 2016년 까지 제주도에서 발생한 446명의 쯔쯔가무시증 대상자들에 대한 분석을 통해 지역별, 시간별 및 인적 특성에 대하여 이해하고 위험요인을 파악하고자 하였다. 지금까지 알려진 쯔쯔가무시증 환자들의 특성은 여성에서 호발하며, 연령분포는 특히 50대 이후의 고령자가 다수를 차지하며 농업 종사자에서 압도적으로 발생 하였고, 이 외에도 추수를 돕거나 등산, 도토리와 같은 열매 채집을 위한 산행한 경험이 있었던 것으로 조사되었다 [8]. 제주 지역에서의 성별비는 남자 45%, 여자 55%로 여자가 더 우세하였으나, 기존에 내륙지방 역학조사에서 보고되던 남자 35%, 여자 65%의 성별비 보다는 격차가 좁은 것으로 확인되었다 [2]. 가을 농작물 수확철인 10-11월에 50-60대 농업종사자에서 많이 발생한 것은 내륙 지방과 유사하였으나, 특이할 만한 점은 과수원 작

업 중 감염되는 비율이 가장 높게 보고되었다. 주된 작업 내용은 감귤 과수업이었다. 이는 내륙지방에서는 논•밭 작업이 주된 감염경로인 것과 차이를 보이며 [9]. 이를 통해 제주 지역에서는 내륙지방과 달리 감귤류를 대표로 한 과수원 농업이 쯔쯔가무시증 발생의 주요 위험 요인임을 확인할 수 있었다. 감귤류의 수확시기는 10월부터 시작하여 12월까지 이어지는데 이는 쯔쯔가무시증의 유행시기와 일치한다. 감귤 과수원 작업시에 어떤 행위가 쯔쯔가무시증 감염의 위험을 높이는지 털진드기의 생활사와 관련된 추가적인 연구가 필요하다 생각된다.

지역 보건소 별 진드기 노출력에 대한 상세활동 조사에서는 각 지역별로 다른 분포를 보였다. 서귀포 동지역과 동부지역은 과수업이, 그 외 지역은 밭농사나 단순 야외활동이 주된 추정 감염경로였는데, 이는 제주도의 농작물 재배 현황이 토양 및 기후 등의 특성에 따라 지역별로 달라짐과 연관이 있다. 제주도의 남•남동 지역인 서귀포 동지역과 서귀포 동부의 남원읍, 표선면은 대표적인 감귤 주산지로 과수업이 주를 이루고, 그 외 지역은 근채류나 양채류 혹은 곡물을 주된 재배 농작물로 하는 밭농사가 주를 이루며, 제주 동지역은 농업 등의 1차 산업 보다는 서비스업 등의 3차 산업이 밀집된 도시화된 지역이다 [10]. 이를 근거로 쯔쯔가무시증에 대한 예방 계획 수립 시, 제주도 내 지역 보건소 별 주된 감염경로

Volume: 39, Article ID: e2017039
https://doi.org/10.4178/epih.e2017039

에 따른 맞춤형 계획이 필요하다 생각된다. 한편 서귀포 동지역과 서귀포 동부지역이 쯔쯔가무시증 발생률이 다른 지역에 비해 높았던 이유가 과수업과 연관이 있음을 가정해 볼 수 있다. 2015년 제주도내 지역 농협 감귤 출하량을 살펴보면 제주도 내 농협 감귤 출하량의 78.8%가 서귀포 동지역 및 서귀포 동부의 남원읍 지역에서 출하된 것으로 확인 되었다 [11]. 이를 뒷받침하듯, 2006년 질병관리본부에서 보고한 심층역학조사 보고서에 따르면 농업 중에서도 과수농사를 짓는 것이 다른 종류의 농업 보다 쯔쯔가무시증의 발생 위험을 유의하게 높임을 증명한 적이 있다 [2].

분석된 기간 중 2016년은 특히 쯔쯔가무시증 발생률이 높게 보고된 해이다. 제주특별자치도 내 대부분의 지역 (한림읍, 안덕면 제외) 에서 전년도 보다 증가된 양상을 보였고, 남원읍과 표선면은 10만명당 발생률이 각각 197.8명과 181.8명까지 치솟았다. 2016년 전국 지역별 10만명당 발생률 평균은 21.5 였고, 다발생 3대 지역은 전남 92.5, 경남 69.4, 전북 55.1이었다 [5]. 먼저 2016년은 전년도의 메르스 사태로 인하여 법정 감염병 신고에 대한 인식 개선과 국가방역체계 개편으로 쯔쯔가무시증을 포함한 제3군 감염병의 신고건수가 39% 증가한 시기이다 [5]. 또한 질병관리본부는 국내 털진드기의 산란기인 8월 평균기온이 털진드기 밀도에

Volume: 39, Article ID: e2017039
https://doi.org/10.4178/epih.e2017039

영향을 주는 요인으로 추정하고 있다 [9]. 여름(특히 8월)에 기온이 높고 상대적으로 건조하면 풀숲 식생이 무성해지고 매개체인 털진드기의 성장과 번식 또한 활발해지기 때문이다. 제주 지역의 2016년도 8월은 북태평양 고기압 및 중국 대륙에서 발달한 열적 고기압의 영향으로 기온이 큰 폭으로 상승하여 평균기온은 28.1도로 기록되었는데 이는 1961년 이래 역대 순위 7위로 기록되었고 [12], 쯔쯔가무시증 발생률 증가에 영향을 끼쳤을 것으로 생각된다.

감염병 예방관리사업이 한정된 자원으로 실질적인 성과를 거두기 위해 해당 감염병의 역학적 특성에 기초한 사업계획이 필요하며, 지역별 특성에 맞는 선택과 집중이 필요하다. 본 연구에서는 제주도에서는 감귤 과수원 농업이 쯔쯔가무시증 발생의 주요 위험 요인이라는 것을 알 수 있었고, 제주도 내 지역별 주요 노출력이 다르므로 제주도에 특이적인 맞춤형 예방 대책 수립이 필요하다 하겠다.

ACKNOWLEDGEMENT

제주특별자치도 6개 지역별 보건소 (제주보건소, 제주동부보건소, 제주서부보건소, 서귀포보건소, 서귀포동부보건소, 서귀포서부보건소) 감염병 담당자 선생님들의 노고에 감사드립니다.

Volume: 39, Article ID: e2017039
https://doi.org/10.4178/epih.e2017039

CONFLICT OF INTEREST

The corresponding author states that there is no conflict of interest.

REFERENCES

1. Korea Centers for Disease Control and Prevention. Infectious disease surveillance year book 2014; 2015 [cited 2017 Aug 1]. Available from: http://www.cdc.go.kr/CDC/info/CdcKrInfo0302.jsp? menuIds=HOME001-MNU1132-MNU1138-MNU0038&fid= 32&q_type=&q_value=&cid=63970&pageNum=(Korean).

2. Korea Centers for Disease Control and Prevention. In-depth investigation report of scrub typhus; 2006 [cited 2017 Jun 1]. Available from: http://www.cdc.go.kr/CDC/contents/CdcKrConten-tLink.jsp?fid=28&cid=1171&ctype=1 (Korean).

3. Chang WH. Scrub typhus. J Korean Med Assoc 1994;37:1400-1407 (Korean).

4. Oaks JS, Ridgway RL, Shirai A, Twartz JC. Scrub typhus: Bulletin No. 21. Kuala Lumpur: United States Army Medical Research Unit, Institute for Medical Research Malaysia; 1983, p. 1-107.

5. Korea Centers for Disease Control and Prevention. Infectious dis-easesurveillance year book 2016; 2017 [cited 2017 Aug 1]. Available from: http://cdc.go.kr/CDC/info/CdcKrInfo0302.jsp?menuIds =HOME001-

MNU1132-MNU1138-MNU0038&fid=32&q_type=&q_value=&cid=75290&pageNum=(Korean).

6. Statistics Korea. 'Demographic change in Jeju province'; 2016 [cited 2017 Jun 1]. Available from: m.kostat.go.kr/board/file_dn.jsp?aSeq=354804&ord=1 (Korean).

7. Korea Meteorological Administration. Climatological feature of Jeju province; 2016 [cited 2017 Jun 1]. Available from: http://www.kma.go.kr/weather/climate/average_regional05.jsp (Korean).

8. Paik SH. A case-control, restrospective study on tsutsugamushi disease occurred in Gyeongju and Pohang provinces, Korea. Korean J Rural Med 2004;29:177-184 (Korean).

9. Korea Centers for Disease Control and Prevention. Epidemiology and management of scrub typhus; 2015 [cited 2017 Jun 1]. Available from: http://www.cdc.go.kr/CDC/contents/CdcKrConten-tLink.jsp?fid=60&cid=65002&ctype=8 (Korean).

10. Choi KJ, Song SH, Kim JS, Lim CW. Estimation of regional agricultural water demand over the Jeju Island. J Environ Sci 2013;22: 639-649 (Korean).

11. Jeju Special Self-Governing Province Citrus Marketing and Shipping Association. Analysis of processing and distribution of Citrus in 2015; 2016 [cited 2017 Jun 1]. Available from: http://citrus.or.kr/board/view.php?btable=bbs&bno=244&p=1&cate=0 (Korean).

12. Korea Meteorological Administration.Characteristics of temperature and precipitation in Jeju province 2016 [cited 2017 Jun 1]. Available from: http://web.kma.go.kr/notify/press/reqional_list.jsp?bid=press2&mode=view&num=8168&page=4&field=&text=&schGrp=제주청 (Korean).

Volume: 39, Article ID: e2017039
https://doi.org/10.4178/epih.e2017039

Table1. Brief regional demographics of Jeju province

	Regions	Area	Total area (km^2)	2011-2015 average population $\times 10^3$(%)	Population density (person /km^2)	Main industry†
City	Jeju	Jeju-si	264	343 (58)	1301	Tertiary
	Seogwipo	Seogwipo-si	254	87 (15)	343	Mix (Primary +Tertiary)
Countryside	Jeju East	Gujwa-eup	185	15 (2)	79	Primary
		Jocheon-eup	151	21 (4)	136	
		Udo-myeon	6	2 (<1)	333	
	Jeju West	Aewol-eup	202	30 (5)	147	Primary
		Hallim-eup	91	20 (3)	215	
		Hangyeong-myeon	79	8 (1)	104	
	Seogwipo East	Seongsan-eup	108	14(2)	130	Primary
		Pyoseon-myeon	135	11 (2)	81	
		Namwon-eup	189	18 (3)	96	
	Seogwipo	Daejeong-eup	79	17 (3)	218	Primary

Volume: 39, Article ID: e2017039
https://doi.org/10.4178/epih.e2017039

West	Andeok-myeon	106	10 (2)	94

†Primary industry includes farming, livestock and fishing. Tertiary industry includes retailing, tourism and administrative service.

Volume: 39, Article ID: e2017039
https://doi.org/10.4178/epih.e2017039

Table 2. Regional incidence of scrub typhus in Jeju province

	Regions	2011		2012		2013		2014		2015		2016		p-value‡
		No. of case	Incidence†	No. of case	Incidence	No. of case	Incidence	No. of case	Incidence	No. of case	Incidence	No. of case	Incidence	
City	Jeju-si	9	2.6	16	4.7	13	3.8	14	4.1	6	1.7	34	9.9	0.006
	Seogwipo-si	12	13.8	10	11.5	8	9.2	9	10.3	13	14.9	16	18.4	
Countryside	Jeju East	1	2.7	7	18.8	8	21.5	4	10.8	7	18.8	10	26.9	0.261
	Jeju West	10	17.4	13	22.6	8	13.9	11	19.2	18	31.4	21	36.6	
	Seogwipo East	18	41.7	18	41.7	13	30.1	16	37.0	13	30.1	60	138.9	0.004
	Seogwipo West	7	25.7	8	29.4	2	7.4	1	3.7	4	14.7	8	29.4	

†per 100,000 person
‡p-value by Mann Whitney U-test for the incidence

Volume: 39, Article ID: e2017039
https://doi.org/10.4178/epih.e2017039

Volume: 39, Article ID: e2017039
https://doi.org/10.4178/epih.e2017039

Table 3. Exposure that may cause scrub typhus by region

	Regions (Total n)	Exposure, n (%)				
		Orchard	Dry field	Hiking	Others*	p‡
City	Jeju (76)	15 (20)	18 (24)	26 (34)†	17(22)	0.000
	Seogwipo (57)	34(60)†	8(14)	10(18)	5(9)	
Countryside	Jeju East (34)	7(21)	13(38) †	8(24)	6 (18)	
	Jeju West (67)	14(21)	28(42)†	13(19)	12 (18)	
	Seogwipo East (117)	76(65)†	20(17)	9(8)	12 (10)	
	Seogwipo West (30)	9(30)	16(53)†	2(7)	3 (10)	

*Others include livestock, forestry, mowing and herb gathering.
†the most common exposure in each region.
‡p-value by Pearson Chi-Square test

Volume: 39, Article ID: e2017039
https://doi.org/10.4178/epih.e2017039

Figure 1

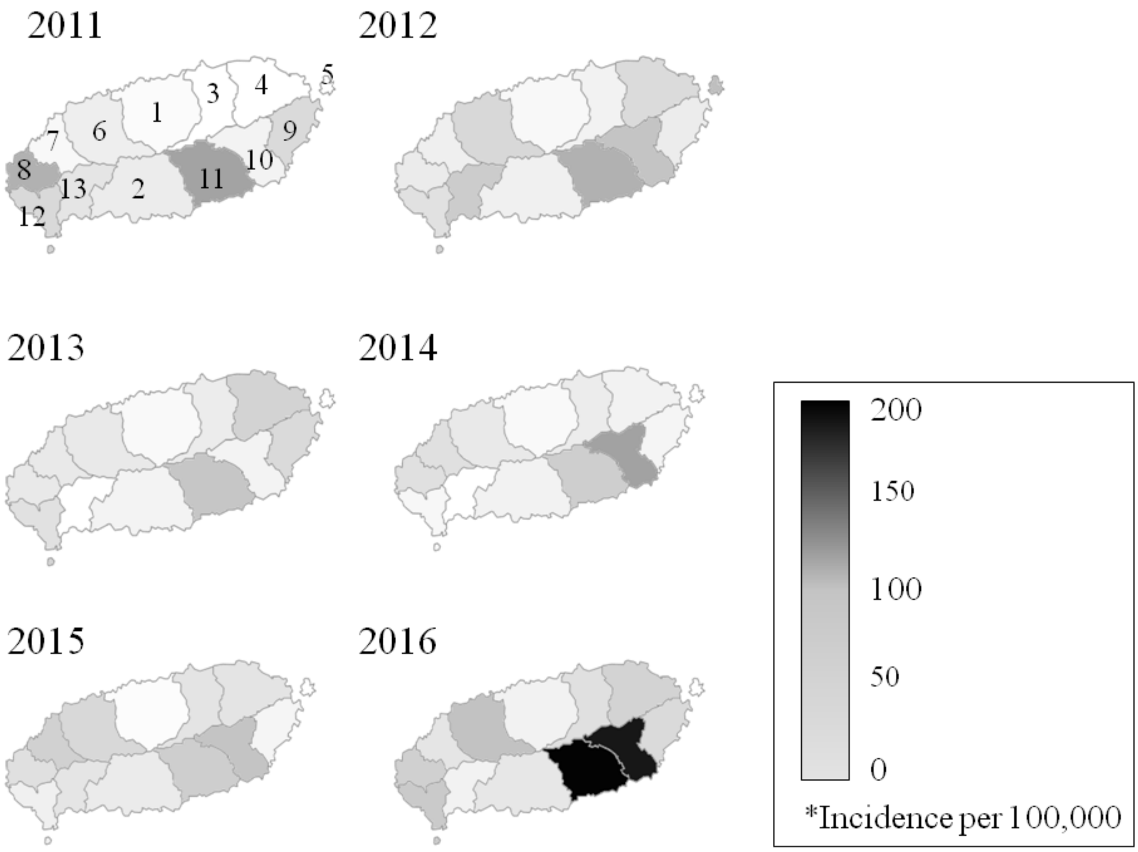

Figure legends

Figure 1. Regional incidence of scrub typhus by year. 1 = Jeju-si, 2 = Seogwipo-si, 3 = Jocheon-eup, 4 = Gujwa-eup, 5 = Udo-myeon, 6 = Aewol-eup, 7 = Hallim-eup, 8. Hangyeong-myeon, 9 = Seongsan-eup, 10 = Pyoseon-myeon, 11 = Namwon-eup, 12 = Daejeong-eup, 13 = Andeok-myeon.

Volume: 39, Article ID: e2017039
https://doi.org/10.4178/epih.e2017039

Figure 2

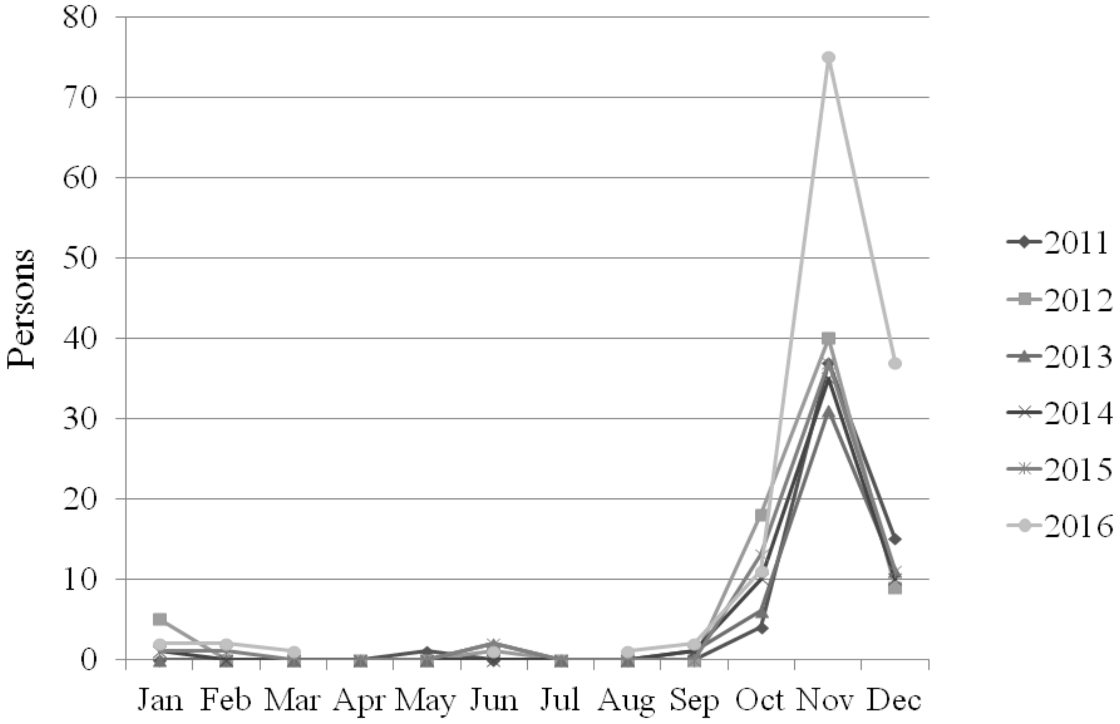

Figure 2. Monthly trend of scrub typhus. The incidence peaked in November.
